# One Size Does Not Fit All: Examining the Effects of Working Memory Capacity on Spoken Word Recognition in Older Adults Using Eye Tracking

**DOI:** 10.3389/fpsyg.2022.841466

**Published:** 2022-04-11

**Authors:** Gal Nitsan, Karen Banai, Boaz M. Ben-David

**Affiliations:** ^1^Baruch Ivcher School of Psychology, Reichman University (IDC), Herzliya, Israel; ^2^Department of Communication Sciences and Disorders, University of Haifa, Haifa, Israel; ^3^Department of Speech-Language Pathology, University of Toronto, Toronto, ON, Canada; ^4^Toronto Rehabilitation Institute, University Health Networks, Toronto, ON, Canada

**Keywords:** speech perception, working memory, aging, word recognition, eye-tracking, visual world paradigm, cognitive hearing science

## Abstract

Difficulties understanding speech form one of the most prevalent complaints among older adults. Successful speech perception depends on top-down linguistic and cognitive processes that interact with the bottom-up sensory processing of the incoming acoustic information. The relative roles of these processes in age-related difficulties in speech perception, especially when listening conditions are not ideal, are still unclear. In the current study, we asked whether older adults with a larger working memory capacity process speech more efficiently than peers with lower capacity when speech is presented in noise, with another task performed in tandem. Using the Eye-tracking of Word Identification in Noise Under Memory Increased Load (E-WINDMIL) an adapted version of the “visual world” paradigm, 36 older listeners were asked to follow spoken instructions presented in background noise, while retaining digits for later recall under low (single-digit) or high (four-digits) memory load. In critical trials, instructions (e.g., “point at the candle”) directed listeners’ gaze to pictures of objects whose names shared onset or offset sounds with the name of a competitor that was displayed on the screen at the same time (e.g., candy or sandal). We compared listeners with different memory capacities on the time course for spoken word recognition under the two memory loads by testing eye-fixations on a named object, relative to fixations on an object whose name shared phonology with the named object. Results indicated two trends. (1) For older adults with lower working memory capacity, increased memory load did not affect online speech processing, however, it impaired offline word recognition accuracy. (2) The reverse pattern was observed for older adults with higher working memory capacity: increased task difficulty significantly decreases online speech processing efficiency but had no effect on offline word recognition accuracy. Results suggest that in older adults, adaptation to adverse listening conditions is at least partially supported by cognitive reserve. Therefore, additional cognitive capacity may lead to greater resilience of older listeners to adverse listening conditions. The differential effects documented by eye movements and accuracy highlight the importance of using both online and offline measures of speech processing to explore age-related changes in speech perception.

## Introduction

A recent report by the World Health Organization (2021) emphasizes the importance of functional ability as a key to healthy aging. It suggests that preserving the abilities to build and maintain relationships and to grow learn and make decisions all promote well-being and healthy aging. These functional abilities depend heavily on successful speech perception. Indeed, difficulties understanding speech are one of the most prevalent complaints among older adults, especially in daily listening situations when listening conditions are not ideal (e.g., Abrams and Farrell, 2011). Although hearing deficits are a main source of difficulty in speech perception (Humes et al., 1994; Humes, 2021), successful speech perception also depends on the interaction of bottom-up hearing related factors and top-down linguistic and cognitive processes (Sommers, 2005; Zekveld et al., 2006; Pichora-Fuller, 2008; Rogers and Peelle, 2021). Furthermore, difficulties in speech perception are also observed among older adults with relatively preserved hearing (Sommers and Danielson, 1999; Fostick et al., 2013; Lash et al., 2013). Our goal is to test whether older listeners with a higher working memory capacity process speech in adverse conditions more efficiently than peers with lower capacity.

Previous studies in cognitive hearing science reported an association between individual differences in cognitive factors and differences in speech perception, even in young and healthy hearing populations. One consistent finding is that these differences are pronounced mainly when using complex testing materials (i.e., sentences, connected discourse comprehension, conversational situations; e.g., Heinrich et al., 2015; Dryden et al., 2017; Meister, 2017). For example, by comparing performance of older listeners across a wide range of speech perception tests differing in complexity, Heinrich et al. (2015) showed that the contribution of cognition increases as the complexity of the speech perception task increases. That is, for older adults, cognitive factors predict sentence perception to a larger extent than single spoken word perception. Of the many cognitive constructs tested, working memory has been widely recognized as related to differences in speech perception abilities, especially in adverse listening condition for older adults (see Akeroyd, 2008; Besser et al., 2013; Dryden et al., 2017 for relevant reviews). In particular, the storage and processing components of working memory play an important role in sentence processing as the listener is required to correctly encode the speech sounds, identify them as words, and then retain the string of words in memory until the sentence is fully heard (Daneman and Carpenter, 1980; Pichora-Fuller et al., 1995; Daneman and Merikle, 1996; Rönnberg et al., 2008). Working memory has also been linked with inhibition of irrelevant information (Awh and Vogel, 2008). The latter is directly related to successful speech perception, where the listener needs to continuously inhibit irrelevant lexical items from his/her mental lexicon to allow correct word recognition. For example, Janse (2012) showed that when speech is presented in background noise, poor inhibitory abilities lead to greater interference by the competing noise which impairs speech perception of older adults.

Contrary to the agreement regarding the association between working memory, aging and spoken sentence processing, only little and mixed evidence is available on this association at the single word level. This is of special importance because lexical ambiguities frequently occur in daily life. For example, cell phones may distort a critical portion of the incoming signal. Consider the sentence “Grandpa! Have you seen the *dog*?” The word *dog* may be mistaken for *doll* (as the two share onset sounds, e.g., see Allopenna et al., 1998; Onset Cohort model, Marslen-Wilson, 1990; Shortlist, Norris, 1994) which can lead to miscommunication with severe consequences on future social participation. Despite these challenges, listeners appear to recognize words with little effort. Moreover, studying the effects of working memory at the single word level has theoretical implications. As spoken sentence processing involves many intervening factors, they may inflate the effects of working memory. Among the abilities necessary to understand sentences are sustained attention for the duration of the sentence and maintaining a running memory of the input to relate what is being heard to what has just been heard and to integrate it with what is about to be heard (Ayasse et al., 2017; Harel-Arbeli et al., 2021). Further, spoken context processing may be more influenced by linguistic experience and vocabulary than the processing of a single spoken word (Stine-Morrow et al., 2006; Borovsky et al., 2012; Ben-David et al., 2015; Kavé and Halamish, 2015). Thus, the aforementioned effects of working memory on the sentence level may reflect other processes. However, if effects are found at a single word level, that would indicate that working memory is involved at very early and basic levels of lexical access.

There is mixed evidence in the literature with regards to the effects of working memory and single spoken word recognition in aging. For example, Heinrich and Knight (2016) found that older adults’ performance in a visual working memory task significantly correlated with their performance on a word in noise (WIN) recognition task, irrespective of the noise level [both in low and high signal to noise ratios (SNRs)]. Gordon-Salant and Cole (2016) found similar results with both young and older adults, correlating auditory working memory capacity, with single-word recognition in noise. Conversely, other studies failed to find this correlation on the single word level. For example, Parbery-Clark et al. (2011) did not find auditory working memory performance to correlate with performance on the WIN test for older adults with and without hearing loss. Similar findings were reported by Smith and Pichora-Fuller (2015) who failed to find a correlation between auditory and visual working memory performance and scores on the WIN test.

A possible explanation for these contradictory findings may stem from the use of offline measures to gauge word recognition (such as accuracy or SNR to achieve 50% recognition). Offline measures test the result of successful (or unsuccessful) word recognition, after the entire word has been heard, processed and a response has been made. It gages the final outcome of the process, and it cannot reveal the early processes underlying online speech processing. Additionally, previous works showed that this association between working memory and word recognition might differ depending on whether verbal or non-verbal measures of working memory are used and the modality of working memory tasks: auditory or visual. There is some evidence to suggest that auditory working memory plays a greater role in speech perception than visual working memory (Baldwin and Ash, 2011; Smith and Pichora-Fuller, 2015; Smith et al., 2016; Kim et al., 2020). Finally, none of the studies listed above tapped cognitive resources while performing speech recognition task, they only measured the correlation between performance on these separate measures. Direct manipulations of the memory load can allow us to better assess the causal relationship between reduced cognitive capacity and spoken word processing in aging.

### The Current Study

In the current study, we examined the role of working memory capacity in spoken word recognition in adverse conditions for older adults. We hypothesized that older listeners with a larger working memory capacity would process speech more efficiently than their peers with a lower capacity; this is tested when speech is presented in noise, with another working memory demanding task performed in tandem. As listeners with lower working memory capacity already have fewer cognitive resources, we expect that the effects of increased load would be especially detrimental for their spoken word processing. This was tested using an adapted version of the eye-tracking “visual world” paradigm, coined the Eye-tracking of Word Identification in Noise Under Memory Increased Load (E-WINDMIL; Hadar et al., 2016; Nitsan et al., 2019). This paradigm was found to have significant test retest reliability for older adults (Baharav et al., 2021). In the E-WINDMIL listeners are instructed to press on one of four objects displayed on the monitor in response to spoken instructions presented in noise. They performed the speech recognition task while retaining for later recall either low (a single spoken digit) or high (four-digits) memory-load. In experimental trials, the named object shares phonology with the name of one of other presented objects. We compared eye-fixations on the named spoken target word, relative to fixations on its phonological competitor, as the word unfolded in time (online). Studies demonstrated that under adverse conditions, spoken word recognition dynamics differ significantly between situations in which the names of the target objects and competitors share an onset and those in which they share an offset in young adults (McQueen and Huettig, 2012; Brouwer and Bradlow, 2016; Hadar et al., 2016), young adults with higher and lower working memory capacity (Nitsan et al., 2019), older adults (Ben-David et al., 2011), and hearing impaired listeners (McMurray et al., 2017). Therefore, the two types of phonological competition will be analyzed separately in the present study, and our analysis will focus on the onset overlap trials.

## Materials and Methods

### Participants

Thirty-eight older adults were recruited from Reichman University’s (IDC) older adult volunteer pool. Of this group, two were excluded due to loss of eye-tracking signal. Thus, the final group for analysis included 36 participants (*M*_age_ = 67.9 years, SD = 3.2, 20 females). All participants met the research inclusion criteria (see Table 1 for details). Participants were paid 35 NIS (approximately $10) for their participation. The number of participants was based on previous studies using a highly similar paradigm (Nitsan et al., 2019; Baharav et al., 2021).

**TABLE 1:** Inclusion criteria for participant recruitment.

	Inclusion criteria
Language background	Proficient Hebrew speakers (no early bilinguals were included) assessed by a self-report and a score within the normal range in the WAIS-III Hebrew vocabulary subtest.
Hearing	Symmetrical air-conduction hearing thresholds expressed as pure tone averages (0.5, 1, and 2 kHz) of ≤25 dB HL in each ear, no reported history of auditory pathology. Audiometric assessment was conducted using a MAICO MA-51 audiometer using standard audiometric procedures in a sound attenuating testing booth.
Vision	Normal or corrected to normal visual acuity and color vision assessed by the Landolt-C charts and the Ishihara charts.
Cognition	Clinically normal scores for their age range on the MoCA cognitive screening test (≥22), and on the forward (≥5) and backward (≥4) digit span subtests (Hebrew version of WAIS-III; *Goodman, 2001).

#### Working Memory

Working memory span was assessed using the forward digit span subtest (Hebrew version of WAIS-III (*Goodman, 2001). To measure the participants’ memory spans, sets of random digits were read aloud at a rate of one per second and they were instructed to repeat them, in the order in which they had been heard. The first list contained two digits, and the number of digits presented for recall increased gradually until the individual was no longer able to recall correctly. Two lists of each length were presented (e.g., two lists of three digits and then two lists of four digits, etc.). A single point was assigned to each list the participant correctly remembered (range of 0–16). Participants were divided into two subgroups based on their digit span scores (range 5–13). The lower-capacity subgroup consisted of 18 participants with a span score of five to nine (*M* = 7.9, SD = 1.1). The higher-capacity subgroup consisted of 18 participants with a span score of 10–13 (*M* = 10.8, SD = 0.89). The two groups did not differ in most individual characteristics, but differed on hearings status, with slightly better audiometric thresholds for the lower-capacity group (see Table 2).

**TABLE 2:** Background information by working memory capacity group.

	Lower capacity	Higher capacity	Group comparison
*N*	18	18	
Age: mean (SD), years	68.5 (2.7)	67.5 (3.6)	*t* = 0.95, *p* = 0.35
Gender: count, females	9	11	χ^2^ = 0.25, *p* = 0.62
Hearing: mean (SD), 0.5, 1, and 2 kHz	15.1 (4.4)	18.7 (4.1)	*t* = 2.5, *p* = 0.02
Years of education: mean (SD)	16.5 (3.2)	16.2 (3.4)	*t* = 0.25, *p* = 0.8
MoCA: mean (SD)	25.5 (1.7)	26.3 (2.5)	*t* = 1.2, *p* = 0.25
Digit span: mean (SD)	7.9 (1.1)	10.8 (0.9)	*t* = 8.5, *p* < 0.001

### Procedure

The experiment was administered individually in a dedicated sound attenuated booth (Iac Acoustics). Participants were seated 60 cm from a computer screen with their head placed in a customized chin rest to stabilize head movement. Each participant’s dominant eye was calibrated to ensure that throughout the course of the trial participants’ online eye-gaze position was recorded. A table mounted SR EyeLink 1000 eye-tracker in the “tower mount” configuration was used (SR Research Ltd., Kanata, ON, Canada). Eye-gaze position was recorded *via* the EyeLink software at a rate of 500 Hz.

**FIGURE 1:**
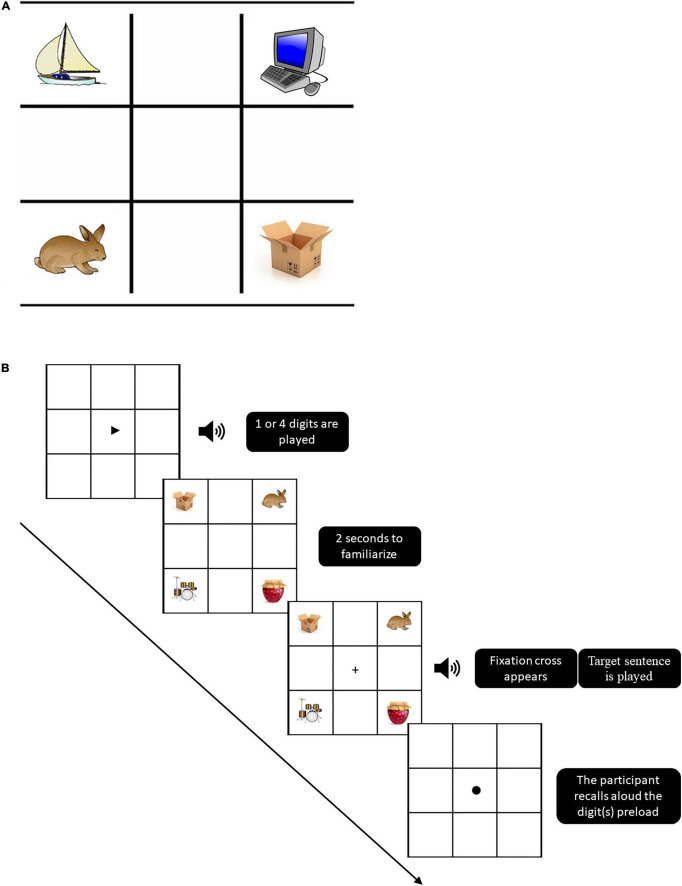
**(A)** Example of an experimental display in Hebrew: the target word, /aʁ.nav/ (rabbit), is represented in the bottom left corner. The onset phonological competitor /aʁ.gaz/ (box), is represented in the bottom right corner. /si.ʁa/ and /max.ʃev/ (boat and computer, respectively) are unrelated distractors. **(B)** Experimental task design: the sequence of displays presented in each trial.

During the experiment, two tasks were presented: spoken word recognition and digit recall (working memory load), conducted in a dual task situation. Trials began with a visual cue of a black “play” triangle centered on the screen, immediately followed by the auditory presentation of the digit(s) preload through headphones, either one digit: low-load condition, or four digits: high-load condition. Participants were told to memorize these digits (in the order presented) for later recall. Then, a 3 × 3 grid with the four images would appear (Figure 1A). Participants were given 2 s to familiarize themselves with the four objects and their position on the computer screen. At the end of these 2 s a flickering fixation cross would appear in the center of the screen, once participants pressed the fixation cross to initiate the trial, the instruction sentence “point at the ___ [target word],” would be presented binaurally *via* the headphones. Selection of a named object was indicated by touching the object picture on the touch screen. Following the participant selection of a stimulus, a visual feedback signal: red highlight for an incorrect answer or green highlight for a correct answer, would appear in the square of the selected image. The visual display would then clear and a visual cue of a black circle would appear in the screen signaling participants to recall aloud the digit(s) preload from the beginning of the trial (Figure 1B illustrates the sequence of displays presented in each trial). The experimenter would then code the response (either correct or incorrect) online. Participants were instructed that speed and accuracy of both the object selection and digit recall were equally important. Participants completed 68 trials split into two trial blocks of the two memory load conditions (Low-load: one digit and High-load: four digits).

Each condition contained 34 trials of which two were practice trials, and 32 were experimental trials. The 32 trials in each condition were split such that 16 were “filler”: target object name did not share any phonology with the surrounding objects, and 16 were “critical” trials in which 8 were phonological onset competitors (e.g., /aʁ.nav/–/aʁ.gaz/ rabbit and box, respectively), and 8 were phonological offset competitors (e.g., /xa.lon/–/ba.lon/ window and balloon, respectively).

### Stimuli

#### Auditory Stimuli

Stimuli were taken from Nitsan et al. (2019), and contained both the object names of the visual stimuli, and the sentence “point at the ___ [target word]” in Hebrew using a plural generic form. All object names were disyllabic. Average target word duration, including the Hebrew article *ha-* (the), was 1078 ms, SD = 91 ms (Nitsan et al., 2019). Considering that the definite article in Hebrew is not a separate word but a prefix, the target word onset was adjusted for each word separately (see Hadar et al., 2016). The root mean square (RMS) intensity was equated across all recorded sentences. Files were mixed with a continuous steady-state speech spectrum noise (for full details, see Ezzatian et al., 2010) at a fixed 0 dB SNR based off of values for discrimination timeline in Ben-David et al. (2012). Stimuli were presented binaurally at 50 dB above individual pure tone average (PTA) *via* a MAICO MA-51 audiometer using TDH 39 supra-aural headphones.

#### Visual Display

On each trial participants were presented with a 3 × 3 grid with four images of objects positioned at the grid corners. The stimuli (images) were previously used by Hadar et al. (2016), Nitsan et al. (2019), and Baharav et al. (2021) studies and were confirmed as clearly identifiable and highly familiar. In all trials one of the four image names represented the spoken target word and a second image’s name was a phonological competitor: sharing the initial syllable (onset sound overlap) or the final syllable (offset sound overlap) with the spoken target word. The remaining two objects presented on screen represented words that were phonologically and semantically unrelated to both the target spoken word and phonological competitor. In critical trials the target word to be recognized was one of the two sound-sharing images. In addition to critical trials, filler trials were used to diminish participant expectation of phonetic resemblance between the words. Objects were presented twice during the experiment, once as a critical trial, and once as a filler trial in which one of the two phonologically “unrelated” items was used as the target word. To prevent implicit spatial learning, object positions on the screen were randomly rotated at each presentation (Farris-Trimble and McMurray, 2013).

### Statistical Analysis

Growth curve analysis (GCA) (Mirman et al., 2008) was used to analyze the time course of fixation from word onset to 1200 ms after word onset (i.e., when target fixations had plateaued). To express listeners’ ability to discriminate the target word from its phonological competitor, we calculated *target discrimination scores* (following: Arnold et al., 2003; Kaiser and Trueswell, 2008; Brown-Schmidt, 2009; Ben-David et al., 2011). To generate the *target discrimination scores*, the proportion of fixations on the competitor was subtracted from the proportion of fixations on the target within 20 ms time bins, starting from the word onset to 1200 ms post word onset. In this measure, the higher the value the better listeners can discriminate the target from its phonological competitor; values approaching zero reflect an inability to discriminate between the target and competitor words. The overall time course of *target discrimination score* was captured with a second-order (quadratic) orthogonal polynomial with fixed effects of capacity group (low vs. high capacity) and working memory load (low vs. high load) on all time terms, and participant random effects on all time terms. The low working memory load condition and the high-capacity group was treated as the reference (baseline) and relative parameters estimated for the high working memory load condition and low-capacity group. These baseline conditions were selected to reflect preserved cognition and the easiest listening condition in this study. The two phonological competition conditions (onset and offset overlap) were modeled separately. Statistical significance (*p*-values) for individual parameter estimates was assessed using the normal approximation.

Offline response accuracy was analyzed using multilevel modeling (Heck et al., 2013) with fixed effects of capacity group (low vs. high capacity) and working memory load (low vs. high load) on response accuracy, participants were included as random effects. All analyses were carried out in SPSS version 25.

## Results

### Onset Overlap – Accuracy of Behavioral Responses

Eye-gaze analysis included only trials in which participants both correctly selected the corresponding object on the visual display (indicating correct spoken word recognition) and correctly recalled the working memory load digits (indicating correct digit recall). Table 3 shows mean accuracy performance across conditions and reflects differential effect of increased load for each working memory capacity group. In the low-capacity group, increasing memory load from one (low load) to four (high load) digits significantly reduced their response accuracy. However, the same increase in task demands did not change response accuracy for the high-capacity group. These differences were confirmed using a multilevel model as detailed in the statistical analysis section. The analysis revealed a main effect of load *F*(1,34) = 13.21, *p* = 0.001 on response accuracy and a significant interaction of load and span *F*(1,34) = 6.60, *p* = 0.015. LSD-corrected pairwise comparisons were conducted to clarify the interaction. It confirmed that the interaction of working memory load and capacity group was due to participants from the low-capacity group being significantly less accurate when a high load was present compared to when a low load present *F*(1,34) = 19.25, *p* < 0.001. In the high-capacity group accuracy did not differ significantly between the two load conditions *F*(1,34) = 0.57, *p* = 0.456.

**TABLE 3:** Mean percentage (and SEs) of trials in which target word was correctly selected and digits were correctly recalled.

	Low WM capacity	High WM capacity
Low WM load	100% (0.0)	97.9% (1.13)
High WM load	83.8% (4.85)	95.1% (2.05)

*Low and high working memory (WM) load, indicate the two preload conditions, one and four digit/s, respectively.*

### Onset Overlap – Eye Gaze

**FIGURE 2:**
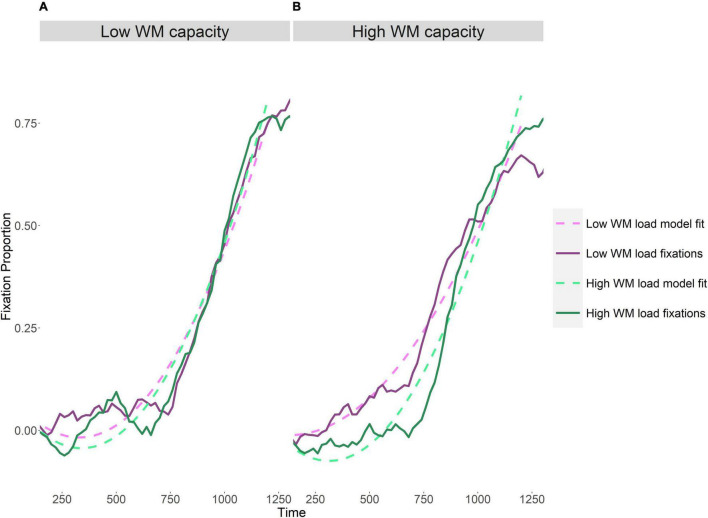
Time-course of *target discrimination scores*. Fixations are shown as a subtraction, with phonological competitor fixations subtracted from the target fixations. The model fits (dashed lines) are plotted along with the observed fixation data (solid lines). Left panel **(A)** show the proportion of fixations for each load condition, one and four digits, respectively, for the low WM capacity group and panel **(B)** show the high WM capacity group.

The data and model fits are shown in Figure 2. Visual inspection of the left panel of Figure 2A shows that for listeners with lower working memory capacity, increasing task demands from low to high working memory load did not change the pattern and rate of target discrimination scores. In contrast, the right panel of Figure 2B indicates that for listeners with higher working memory capacity, increasing the working memory load delayed processing, suggesting less efficient spoken word processing. The results of the analysis as shown in Table 4 confirm these observations. The analysis shows a significant effect of capacity group on the intercept and all polynomial time terms (linear and quadratic), suggesting that the rate of accumulating evidence from the unfolding spoken word differs between the two capacity groups. Working memory load was also found to have a significant effect on the linear and quadratic time terms, again suggesting a difference in evidence accumulation. Most importantly, the interaction between working memory load and capacity group on the linear and quadratic time terms was found to be significant.

**TABLE 4:** Results of growth curve analysis (GCA) – onset overlap.

	Term	Estimate	SE	*t*-Value	*p*
Participant group (WM capacity)	Intercept	0.094	0.041	2.28	0.025
	Linear	−0.502	0.105	−4.79	< 0.001
	Quadratic	0.004	<0.001	4.26	< 0.001
Working memory load	Intercept	0.037	0.027	1.37	0.170
	Linear	−0.541	0.105	−5.16	< 0.001
	Quadratic	0.001	<0.001	5.62	< 0.001
Participant group (WM capacity) × working memory load	Intercept	−0.041	0.038	−1.07	0.287
	Linear	0.429	0.148	2.89	0.004
	Quadratic	−0.001	<0.001	−2.86	0.004

A follow up model conducted separately for each capacity group revealed the source of this interaction (Table 5). In the low-capacity group, no significant effect of working memory load was evident; whereas in the high-capacity group the effect of working memory load on the linear and quadratic time terms was significant. The significant effect of working memory load on the linear term indicates a steeper slope, faster accumulation of evidence, under low working memory load. The effect of working memory load on the quadratic term further showcases a difference in the change in the rate of evidence accumulation between the two load conditions.

**TABLE 5:** Results of growth curve analysis (GCA) conducted separately for each WM capacity group.

	Term	Estimate	SE	*t*-Value	*p*
Low-capacity group – working memory load	Intercept	−0.004	0.029	−0.12	0.902
	Linear	−0.112	0.112	−0.10	0.319
	Quadratic	0.000	0.000	1.47	0.141
High-capacity group – working memory load	Intercept	0.037	0.025	1.49	0.137
	Linear	−0.541	0.967	−5.59	<0.001
	Quadratic	0.000	<0.001	6.09	<0.001

In sum, eye-movement analyses of onset overlap trials indicate that for the higher working memory capacity group, an increase in working memory load slowed spoken word processing. This slowdown was not evident for the lower working memory capacity group.

The same analyses conducted for the onset overlap trials were replicated for the offset overlap condition. The effects noted in the eye-gaze for the onset overlap condition were not found in the offset overlap, but for the effect of working memory capacity group. Analysis of accuracy of behavioral responses in the offset overlap revealed that increasing memory load from one to four digits significantly reduced listeners’ response accuracy regardless of span group membership. Additionally, is shows that overall listeners from the high-capacity group had higher response accuracy compared to listeners from the low-capacity group. The low-capacity group had a greater reduction in response accuracy compared to the high-capacity group. The full analysis is provided in Appendix A.

## Discussion

We investigated the efficacy with which older adults with different working memory capacities process a spoken word in adverse conditions. Both online (eye-tracking) and offline (behavioral response accuracy) measures for spoken word recognition were used. Consistent with our hypothesis, we report that increasing task demands had different effects on listeners with higher vs. lower working memory capacity when the target and competitor shared onset sounds. Overall, listeners with higher working memory capacity were able to maintain their offline response accuracy at maximal performance even when they were asked to retain four digits for later recall instead of only one digit (high and low working memory load, respectively). However, this increase in working memory load had slowed down their online spoken word processing, suggesting less efficient processing at the single word level. For listeners with lower working memory capacity, increasing task demands significantly reduced offline recognition accuracy (from ∼100 to ∼80%), with no effect on online word processing. In the offset sound sharing condition, increasing memory load from one to four digits significantly reduced listeners’ offline response accuracy regardless of their working memory capacity without affecting their online processing.

Our results present a clear support for the involvement of cognition, and more specifically working memory, in speech perception for older adults, even in the processing of a single spoken word. The literature to-date is inconsistent with regards to this question. Some studies on older adults observed correlations between working memory scores and recognition of single words in noise (Gordon-Salant and Cole, 2016; Heinrich and Knight, 2016) while others did not (Parbery-Clark et al., 2011; Smith and Pichora-Fuller, 2015). The present study has the distinct advantage of directly manipulating memory load, testing the effect of reduced cognitive resources on spoken word processing in aging. By varying the number of digits to be remembered (one vs. four digits) we were able to temporarily deplete spare cognitive capacity while listeners performed a speech recognition task in noise. This momentary depletion led to changes in offline word recognition (for the lower-capacity group) and in online word processing (for the higher-capacity group). Note, if we were to test offline word recognition only, results would suggest that cognitive depletion mainly affects individuals with already low cognitive reserve. Indeed, previous works showed that increasing working memory load impairs language processing for clinical populations with reduced working memory capacity, such as people with aphasia, to a larger extent than for neurologically intact adults (Martin et al., 2012; Obermeyer et al., 2021). By using online measures, the current study shows the intricate effect of working memory depletion already at the single word level, even for individuals with larger cognitive reserves. Therefore, accessing and retrieving words from the mental lexicon when the input is degraded may require some available working memory resources even in healthy older adults with no signs of cognitive impairment. This link between cognition and speech processing in adverse listening conditions may stem from correlated activity across different brain regions. Indeed, spoken language processing rely on the joint activation of multiple cortical subsystems and several attempts were done to estimate its effectiveness by measuring cortical evoked responses (Gow, 2012). For example, Kim et al. (2021) suggested that changes in left supramarginal gyrus activity may be used as an independent predictor for speech processing efficiency.

In our analysis we found a differential effect of increasing working memory load for individuals with higher and lower working memory capacities. While increased load impaired offline accuracy for individuals with lower capacity, it affected online processing efficiency for individuals with higher capacity. According to the Framework for Understanding Effortful Listening (FUEL; Pichora-Fuller et al., 2016) speech processing depends on deployment of cognitive resources and therefore might be affected by differences in maximal capacity, especially under increased perceptual effort conditions such as in the presence of background noise and working memory load. It is possible that the listeners with lower working memory capacity were already using all their available resources in the low load condition in order to achieve maximal performance (100% accuracy). In other words, their online spoken word processing efficiency reflects their maximal ability. When facing increased task demands, they had no more available resources to allocate. Thus, with the same (maximal) word processing efficiency, as indicated by the online measures, their offline accuracy was significantly reduced. It is important to note that our analysis included only trials in which participants both correctly recognized the spoken word and correctly recalled the working memory load digits. Removing incorrect trials arguably removes the most challenging trials from the analysis which might lead to an under-estimation of the effects of increased load on individuals with lower working memory capacity. In contrast, listeners with higher working memory capacity were not using all their available resources in the low load condition. Consequently, when working memory load increased they still had some spare available resources to allocate to maintain their performance. But this came with a cost of slower online word processing.

Our results might be interpreted in light of the Ease of Language Understanding (ELU) model (Rönnberg et al., 2013). According to the model, understanding speech in adverse conditions is possible by drawing on central cognitive resources, mainly identified with working memory resources to compensate for the loss of automatic matching between the input and lexical representations when the input is degraded. Consistent with our findings, this model predicts that individuals with higher working memory capacity will be able to allocate these resources to maintain their offline performance. Changes in online processing could reflect either input degradation or the increased effort associated with the loss of automated word recognition.

In contrast to previous studies that relied on offline measures alone, the present study employed also online measures to track word processing as the acoustic signal unfolded over time. Standard measures of offline spoken word recognition accuracy do not capture the cost associated with maintaining a good level of performance. Our results highlight the importance of using both online and offline measures of speech processing to explore age-related changes in speech perception. The current study joins other studies that effectively used the visual world paradigm as a gauge of speech processing in adverse listening conditions (McQueen and Huettig, 2012; Helfer and Staub, 2014; Brouwer and Bradlow, 2016; McMurray et al., 2017). For example, McMurray et al. (2017) demonstrated that listeners with normal hearing process speech in a similar manner to that of cochlear implant users, when listening to severely degraded speech. In exploring the temporal dynamics of word recognition, authors could not only gauge the timing of target word recognition, but also determine the level and type of lexical competition that listeners were experiencing. Recent work from our lab also demonstrated that group-differences related to working memory load that were obscured in offline measures (e.g., accuracy) were uncovered when gaging online eye-tracking measures (Hadar et al., 2016; Nitsan et al., 2019; Harel-Arbeli et al., 2021).

### Conclusions and Future Studies

The present data illustrate the differential effect of increasing task demands on spoken word recognition by listeners with higher vs. lower working memory capacity. Our findings suggest that additional cognitive capacity may lead to greater resilience of older listeners to adverse listening conditions. Future studies may wish to examine this paradigm using different types of adverse listening condition such as fast speech. Understanding accelerated speech is another predominant complaint among elderly listeners but little is known about its time course (Humes and Dubno, 2010; Banai and Lavie, 2020; Rotman et al., 2020). Studies should also consider carefully controlling for the possible effects of stress and stereotype threat on hearing assessments (Ben-David et al., 2018; Nagar et al., 2022). Another path for investigation is testing these findings in clinical populations with cognitive decline (noting the difficulties in adaptation, Tziraki et al., 2017) or hearing aids and cochlear implant users to better tailor hearing rehabilitation expectations (e.g., Taitelbaum-Swead et al., 2022). Future studies may also choose to further examine the effects of working memory load and span on brain activity involved in speech processing in aging.

## Data Availability Statement

The raw data supporting the conclusions of this article will be made available by the authors, without undue reservation.

## Ethics Statement

The studies involving human participants were reviewed and approved by the School of Psychology Review Board, Reichman University, and by the Ethics Committee of the Faculty of Social Welfare and Health Sciences, University of Haifa. The participants provided their written informed consent to participate in this study.

## Author Contributions

GN, KB, and BB-D wrote the manuscript. GN was responsible of the analysis and interpretation of the data. KB contributed to the conceptualizing of the research question and interpreting the results. BB-D was responsible of the design of the paradigm, the analysis, and the interpretation of the results. BB-D was the corresponding author and the study was conducted in his lab. All authors had a prominent intellectual contribution to the study, are accountable for the data and approved the final version of the manuscript.

## Conflict of Interest

The authors declare that the research was conducted in the absence of any commercial or financial relationships that could be construed as a potential conflict of interest.

## Publisher’s Note

All claims expressed in this article are solely those of the authors and do not necessarily represent those of their affiliated organizations, or those of the publisher, the editors and the reviewers. Any product that may be evaluated in this article, or claim that may be made by its manufacturer, is not guaranteed or endorsed by the publisher.

## Appendix A. Full Analysis of the Offset Overlap Condition

### Eye Gaze

Unlike the onset overlap condition, the analysis of offset overlap trials showed only an effect of span group on the linear and quadratic time terms, suggesting differential online word processing between span groups. Table A1 summarizes the results of the analysis.

**TABLE A1:** Results of growth curve analysis (GCA) – offset overlap.

	Term	Estimate	SE	*t*-Value	*p*
Participant group (WM capacity)	Intercept	−0.053	0.039	−1.37	0.174
	Linear	0.333	0.099	3.36	< 0.001
	Quadratic	<0.001	<0.001	−3.13	0.002
Working memory load	Intercept	−0.013	0.026	−0.51	0.607
	Linear	0.064	0.099	0.644	0.519
	Quadratic	<0.001	<0.001	−0.78	0.438
Participant group (WM capacity) × working memory load	Intercept	0.004	0.036	0.11	0.915
	Linear	−0.132	0.140	−0.94	0.347
	Quadratic	<0.001	<0.001	1.09	0.275

### Accuracy of Behavioral Responses

Eye-gaze analysis included only trials in which participants both correctly selected the corresponding object on the visual display (indicating correct spoken word recognition) and correctly recalled the working memory load digits (indicating correct digit recall). The analysis indicated a main effect of load *F*(1,34) = 34.23, *p* < 0.001 and span group *F*(1,34) = 6.83, *p* = 0.013 on response accuracy. These two effects suggest that increasing memory load from one to four digits significantly reduced listeners’ response accuracy regardless of span group membership. Additionally, is shows that overall listeners from the high span group had higher response accuracy (*M* = 95.83 vs. *M* = 89.48) compared to listeners from the low span group. The two effects interacted significantly *F*(1,34) = 5.59, *p* = 0.024. LSD-corrected pairwise comparisons revealed that increasing memory load yielded greater reduction in response accuracy in the low span group *F*(1,34) = 33.75, *p* < 0.001 compared to the high span group *F*(1,34) = 6.08, *p* = 0.019 as shown in Table A2.

**TABLE A2:** Mean percentage (and SEs) of trials in which target word was correctly selected and digits were correctly recalled.

	Low WM capacity	High WM capacity
Low WM load	99.3% (0.69)	100% (0.0)
High WM load	79.7% (4.08)	91.7% (2.48)

*Low and high WM, indicate the two preload conditions, one and four digit/s, respectively.*
